# Comparison of gene co-networks analysis provide a systems view of rice (*Oryza sativa* L.) response to *Tilletia horrida* infection

**DOI:** 10.1371/journal.pone.0202309

**Published:** 2018-10-29

**Authors:** Aijun Wang, Xinyue Shu, Xianyu Niu, Wenjuan Zhao, Peng Ai, Ping Li, Aiping Zheng

**Affiliations:** 1 Rice Research Institute of Sichuan Agricultural University, Chengdu, Sichuan, China; 2 Key laboratory of Sichuan Crop Major Disease, Sichuan Agricultural University, Chengdu, Sichuan, China; 3 Key Laboratory of Southwest Crop Gene Resource and Genetic Improvement of Ministry of Education, Sichuan Agricultural University, Ya’ an, Sichuan, China; Fujian Agriculture and Forestry University, CHINA

## Abstract

The biotrophic soil-borne fungus *Tilletia horrida* causes rice kernel smut, an important disease affecting the production of rice male sterile lines in most hybrid rice growing regions of the world. There are no successful ways of controlling this disease and there has been little study of mechanisms of resistance to *T*. *horrida*. Based on transcriptional data of different infection time points, we found 23, 782 and 23, 718 differentially expressed genes (fragments per kilobase of transcript sequence per million, FPKM >1) in Jiangcheng 3A (resistant to *T*. *horrida*) and 9311A (susceptible to *T*. *horrida*), respectively. In order to illuminate the differential responses of the two rice male sterile lines to *T*. *horrida*, we identified gene co-expression modules using the method of weighted gene co-expression network analysis (WGCNA) and compared the different biological functions of gene co-expression networks in key modules at different infection time points. The results indicated that gene co-expression networks in the two rice genotypes were different and that genes contained in some modules of the two groups may play important roles in resistance to *T*. *horrida*, such as DTH8 and OsHop/Sti1a. Furthermore, these results provide a global view of the responses of two different phenotypes to *T*. *horrida*, and assist our understanding of the regulation of expression changes after *T*. *horrida* infection.

## Introduction

Rice kernel smut (RKS), caused by the soil-borne basidiomycete fungus *Tilletia horrida*, is the most devastating disease during the production of rice male sterile lines in most hybrid rice growing regions of the world [1]. RKS can lead to yield losses of 5–20% in general and even 50% in severe cases [2], it is an increasing threat to rice cultivation in Asia, Oceania, Europe, America, and Africa [3,4]. Although the quantitative resistant of rice male sterile lines to the RKS have been identified [5], complete resistance or immunity have not been found. Despite the importance of RKS, there have been few studies of *T*. *horrida*, partly because as a biotrophic pathogen, cultured it on artificial media is extremely difficult and there are very few effective resistant rice cultivars [6].

With the development of sequencing techniques, transcriptome analysis and genomics have promoted the study of the molecular mechanisms of disease resistance and tolerance to stress [7]. These processes often involve multiple biological function genes; however, the analysis of multiple function genes using traditional biological methods is difficult [8]. In previous studies, the identification of candidate genes that related to biological phenotype was mainly based on comparing gene expression levels of differentially expressed genes between different experimental groups [9–11]. This method mainly focused on single model research, nowadays, the study of molecular interactions in dynamic network changes has been extended [12]. Biological networks provide valuable platforms for understanding system-level response to different biological phenotypes, such as disease resistance [13–15]. The main biological networks include signaling networks, protein–protein interaction networks, metabolic networks, and gene co-expression networks. Of these four biological networks, gene co-expression networks analysis has many advantages [16].

Gene co-expression networks are increasingly used to explore the functionality of genes involved in biological phenotypes, in which nodes represent genes and edges are used to connect two correlated genes to express the relationship between them [17, 18]. Previous studies have shown that co-expression network could identify how gene expression is regulated and involved in each different phenotype based on a more systematic perspective; they can also explore the relationship of gene functionality to phenotype by observing pairwise correlations between gene transcripts [19, 20]. Weighted gene co-expression network analysis (WGCNA) is a systems biology approach that describes the correlation of modules according to microarray analysis, and showed how modules of highly correlated genes facilitate an understanding of multiple gene expression networks instead of individual genes [21]. Furthermore, related functionally enriched modules are implicated in complex diseases and many candidate biomarkers or genes have been successfully identified using this method, e.g. wheat resistance responses to powdery mildew [22–28].

We set out to clarify which systemic functions of cellular components are important in resistance to *T*. *horrida* in rice, and to understand the change in gene expression in resistant and susceptible host backgrounds. We used the rice male sterile line Jiangcheng 3A, which is resistant to kernel smut, and a susceptible rice male sterile line, 9311A, as host plants to investigate *T*. *horrida* infection. We set out to identify the genes and major modules related to resistance against *T*. *horrida* infection and to analyze the genes that are involved in resistance co-expression networks using WGCNA. Based on transcriptomics data, we compared the gene co-expression networks of these two rice varieties after infection with *T*. *horrida*. Our results explain the changes in gene expression after *T*. *horrida* infection by providing an efficient gene co-expression network in two rice varieties with different defensive phenotypes. These findings promote an understanding of the resistance mechanism to RKS, which aid our attempts control this destructive disease.

## Materials and methods

### Strains and rice varieties

The *T*. *horrida* strain JY-521 was isolated from a rice field infected with RKS in Sichuan province by the spore suspension method [29]. The resistant rice male sterile line “Jiangcheng 3A” has been previously identified through inoculation trials with *T*. *horrida* during 2014–2016 [5], and “9311A” is a highly susceptible standard line. Jiangcheng 3A and 9311A seeds were obtained from the Department of Rice Research Institute of Sichuan Agricultural University.

### The transcriptional sample and RNA-Seq

The *T*. *horrida* JY-521 monoconidial cultures were grown in potato dextrose broth in an incubator shaker at 150 r. p. m. and 28°C for 7 d. About 1mL of conidial suspension (10^6^ conidia mL^-1^) was injected into the immature kernel of field grown rice plants at the booting stage 5–7 d before heading. Because the temperature was low in the late afternoon and better infection, so this was done in this time. Immature kernels were harvested at 8, 12, 24, 48, and 72 h after *T*. *horrida* JY-521 infection, kernels inoculated with 1ml of sterilized distilled water for 8 h served as the control (infection 0 h) for each cultivar, and three biological replicates per processing time point, were frozen immediately in liquid nitrogen and subsequently kept at -80°C for RNA isolation. A total of 36 samples were obtained for RNA-Seq in this study.

RNA-Seq libraries were constructed using NEBNext Ultra RNA Library Prep Kit for Illumina (NEB, USA), according to the manufacturer’s instructions. Sequencing was performed using an Illumina Hiseq platform to generate 125 bp paired-end reads. Reads containing adapt stretched of -Ns and read with low quality score were removed from the raw data. The reference genome of Nipponbare rice and gene model annotation files (Rice Annotation Project) were downloaded from the genome website directly (ftp://ftp.ensemblgenomes.org/pub/plants/release_36/fasta/oryza_indica/dna/). Index of the reference genome was built using Bowtie v2.2.3, and paired-end reads were aligned to the reference genome using TopHat v2.0.12 [30]. HTSeq v0.6.1 was used to count the reads numbers mapped to each gene [31]. Then, the number of fragments per kilobase of transcript sequence per million (FPKM) of each gene was calculated based on the length of the gene and the number of read counts mapped to that gene.

### Analysis of differential expression genes

Differential expression analysis of two conditions (three biological replicates per condition) was performed in R using the DESeq package (1.18.0) [32]. DESeq provides statistical routines for determining differential expression in digital gene expression data using a model based on negative binomial distribution. The resulting P-values were adjusted using the Benjamini and Hochberg’s approach for controlling the FDR. Genes with adjusted P <0.05 were designated as differentially expressed. The normalized data were used in our network analysis.

### qRT-PCR

First strand cDNA was synthesized from total RNA using Transcriptor First-Strand cDNA Synthesis Kit (Roche, Indianapolis, IN, USA). The cDNA samples were then subjected to qRT-PCR using Bio-Rad CFX96 Real-Time PCR System (Foster City, CA, USA), according to the manufacturer’s instructions. The PCR reactions were prepared in a 20μL volume, containing 3 μL cDNA and 0.8 μL each of the forward and reverse gene specific primers, each PCR was replicated three times. The Ubiquitin (UBQ) gene was used as an internal control for data normalization. The expression levels of gene were calculated using the 2^-ΔΔCt^ algorithm. The amount of *T*. *horrida* biomass in rice kernels was quantified based on the abundance 18S rRNA of *T*. *horrida* relative to that of rice [10]. Primers used for qRT-PCR are listed in S1 Table.

### Network construction

Co-expression networks were constructed using the WGCNA (v1.47) package in R [17]. The gene co-expression network is a scale-free weighted gene network with the significant feature that most nodes have only a few connections, and only a few nodes having a large number of connections [33]. To satisfy the preconditions of scale-free network distribution, we selected minimum power values when correlation coefficients were higher than 0.8 as analysis values for further study. We evaluated power values from 1 to 20, and the corresponding correlation coefficients of the adjacent genes were calculated. We selected power values = 9 for 9311A and power values = 8 for Jiangcheng 3A to construct the co-expression networks (S1 and S2 Figs). Based on the above analysis, we constructed a WGCNA to subdivide thousands of genes into several modules. To find biologically significant modules, module eigengenes were used to calculate the correlation coefficient with 36 rice kernel samples and then calculation were made of their association with the two genotypes (Jiangcheng 3A and 9311A). The results were presented using heat maps based on correlation coefficients, with a deeper color representing a higher correlation.

### Functional analysis of module genes

In order to analyze the biological functions of genes in each module, GO and KEGG pathway enrichment analysis were conducted. Significantly enriched GO terms and pathways in genes in a module compared to the background were defined by hypergeometric tests and a threshold of false discovery rate (FDR) less than 0.05.

### Module and hub gene selection

In each module, the hub genes have important biological significance and a bond that to united other genes [34]. In order to compare the difference in resistance between Jiangcheng 3A and 9311A, the early and later stage modules that had high correlation were selected and the hub genes with a high degree of connection in these modules were identified. Significantly correlation module and phenotype were defined by hypergeometric tests and P <0.01 as correlation. To further explore interactions among genes in each module, we selected the first 50 genes (hub genes) with the highest connectivity within the corresponding module to draw the gene network using Cytoscape_3.3.0.

In order to identify the difference in disease resistance between Jiangcheng 3A and 9311A, we considered the top 1000 genes that had the highest connectivity in the two male sterile lines as core genes. In addition, we obtained 3,131 disease resistance genes using Oryzabase (https://shigen.nig.ac.jp/rice/oryzabase/), among them 2,482 appeared in Jiangcheng 3A and 2,484 in 9311A (S2 and S3 Tables). We extracted core resistance genes from 1,000 core genes in Jiangcheng 3A and 9311A, respectively, and mapped the co-expression network.

## Results

### Quantification of *T*. *horrida* growth in resistant and susceptible rice male sterile lines

**Fig 1:**
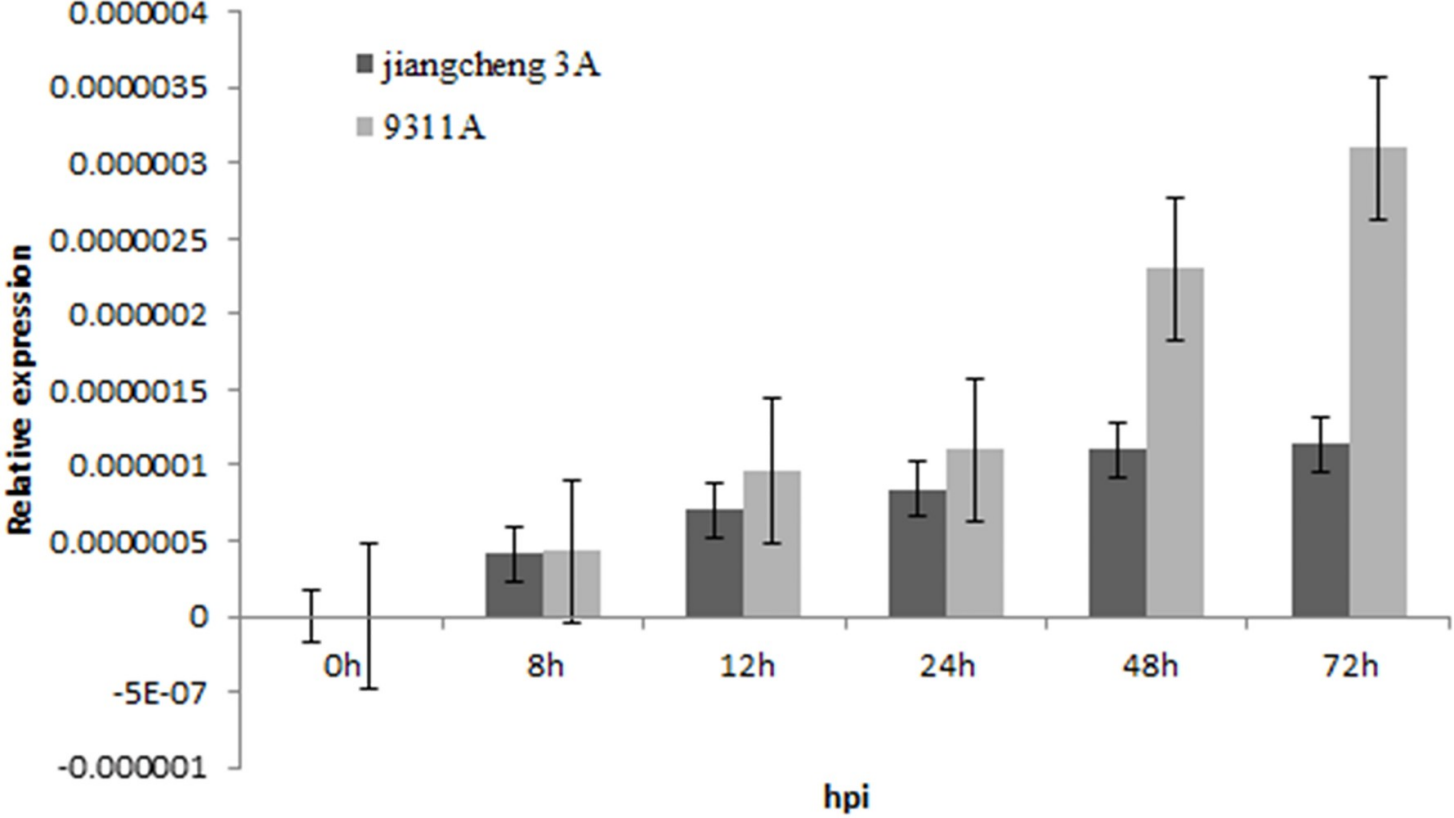
Relative *T*. *horrida* fungal abundance determined by qRT-PCR analysis of *T*. *horrida* 18S rRNA sequence in samples harvested at 0, 8, 12, 24, 48, and 72h post inoculation.

To identify the colonization of *T*. *horrida* in immature kernels of two rice male sterile cultivars, we quantified the relative amount of fungal biomass in immature kernels of Jiangcheng 3A and 9311A using qRT-PCR during the early stages of infection, specifically from 8h to 72h post inoculation. *T*. *horrida* spores were detected in both cultivars 8 h after infection. However, the biomass of *T*. *horrida* in 9311A immature kernels increased by 12 h, was higher than that in Jiangcheng 3A immature kernels (Fig 1).

### Analyses of global genes

After *T*. *horrida* infection, 23,782 and 23,718 expression genes were identified at different times in Jiangcheng 3A and 9311A, respectively, the fragments per kilobase of transcript sequence per million (FPKM) were higher than 1 for all 36 samples. The rice gene co-expression network was established based on expression data of these genes in Jiangcheng 3A and 9311A using WGCNA, the profile of same expressed trend genes were identified and assigned to different modules according to a previously used method, one colors representing one module [35, 36]. The differential expression data after *T*. *horrida* infection could reflect different biological processes at different time points. So, in order to clarify the regulation pattern of biological process and function association in rice resistant to *T*. *horrida*, we constructed a reduced network structure using transcriptome data of different infection time points.

### qRT-PCR validation of expression genes

To verifiy the RNA-Seq data, the rice pathogenesis-related proteins OsPR1a, OsPR1b, and OsPR10a, a member of the WRKY gene superfamily in rice, OsWRKY24 and OsWRKY70 were selected for validation. In addition, several genes that related to resistance-*T*. *horrida*, such as DTH8 and OsHop/Sti1a were also selected for validation. The threshold cycle (Ct) values of each gene were normalized relative to those of the Ubiquitin gene (internal control). The expression patterns of these gene obtained using qRT-PCR analysis were compared with Illumina RNA-Seq data (S3 Fig). Results showed that the trend of qRT-PCR data was consistent with that of Illumina data, suggesting that the Illumina RNA-Seq data were reliable.

### Network construction

**Fig 2:**
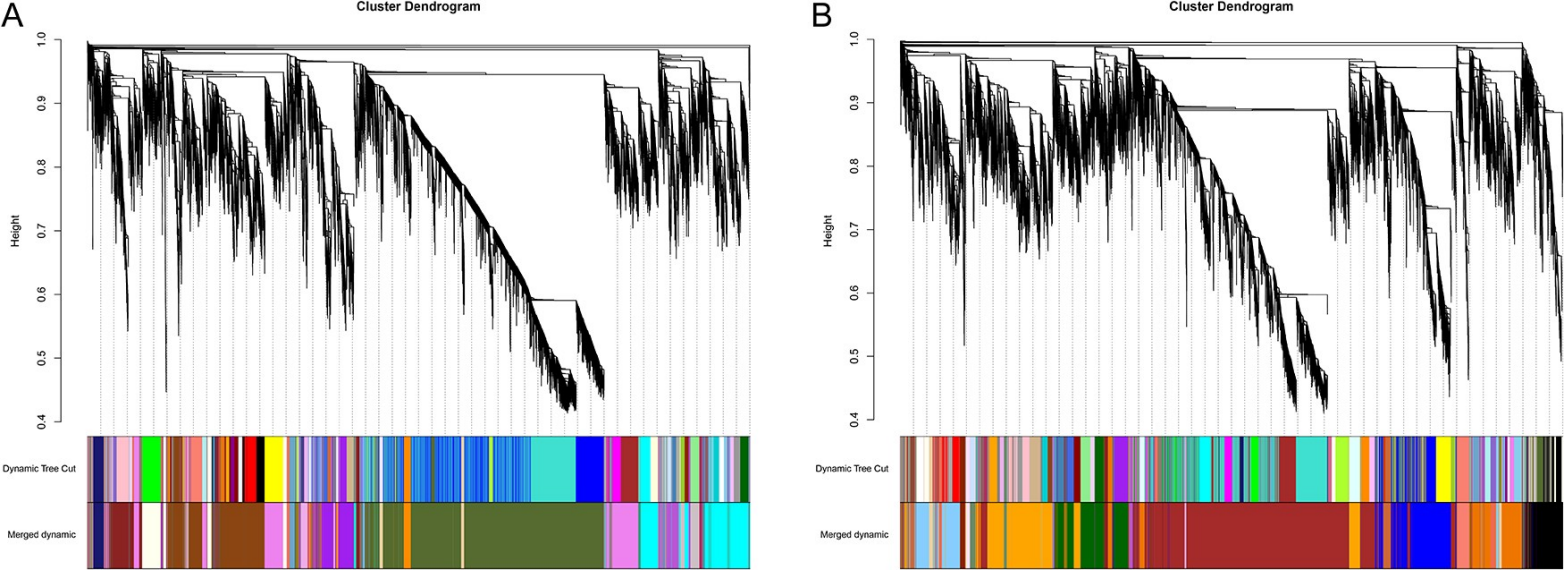
WGCNA of genes in kernel tissues of Jiangcheng 3A (A) and 9311A (B) after *T*. *horrida* infection. Hierarchical cluster trees show the co-expression modules identified by WGCNA.

WGCNA of two rice male sterile lines was performed, and the results showed that 19 distinct modules were generated in Jiangcheng 3A and 9311A through a hierarchical clustering tree, in which each tree branch represented a module, with a leaf in the branch as one gene (Fig 2). Sets of genes of common expression patterns at different time points were identified in the same modules. The number of genes in each module ranged from 91 to 9,392 for Jiangcheng 3A (Fig 2A) and from 69 to 8,982 for 9311A (Fig 2B) and shown that division of modules in two rice male sterile lines was different. Module-Trait Relationships (MTRs) of each module at different points was also different. These genes involved in different modules that were up-regulated or down-regulated at different time points after *T*. *horrida* infection. The results indicated that some modules were positively or negatively correlated with *T*. *horrida* infection. Subsequently, some genes contained in modules (MTRs>0.7) were selected for further analysis based on their intra-module connectivity.

### Modules associated with differences between Jiangcheng 3A and 9311A after *T*. *horrida* infection

**Fig 3:**
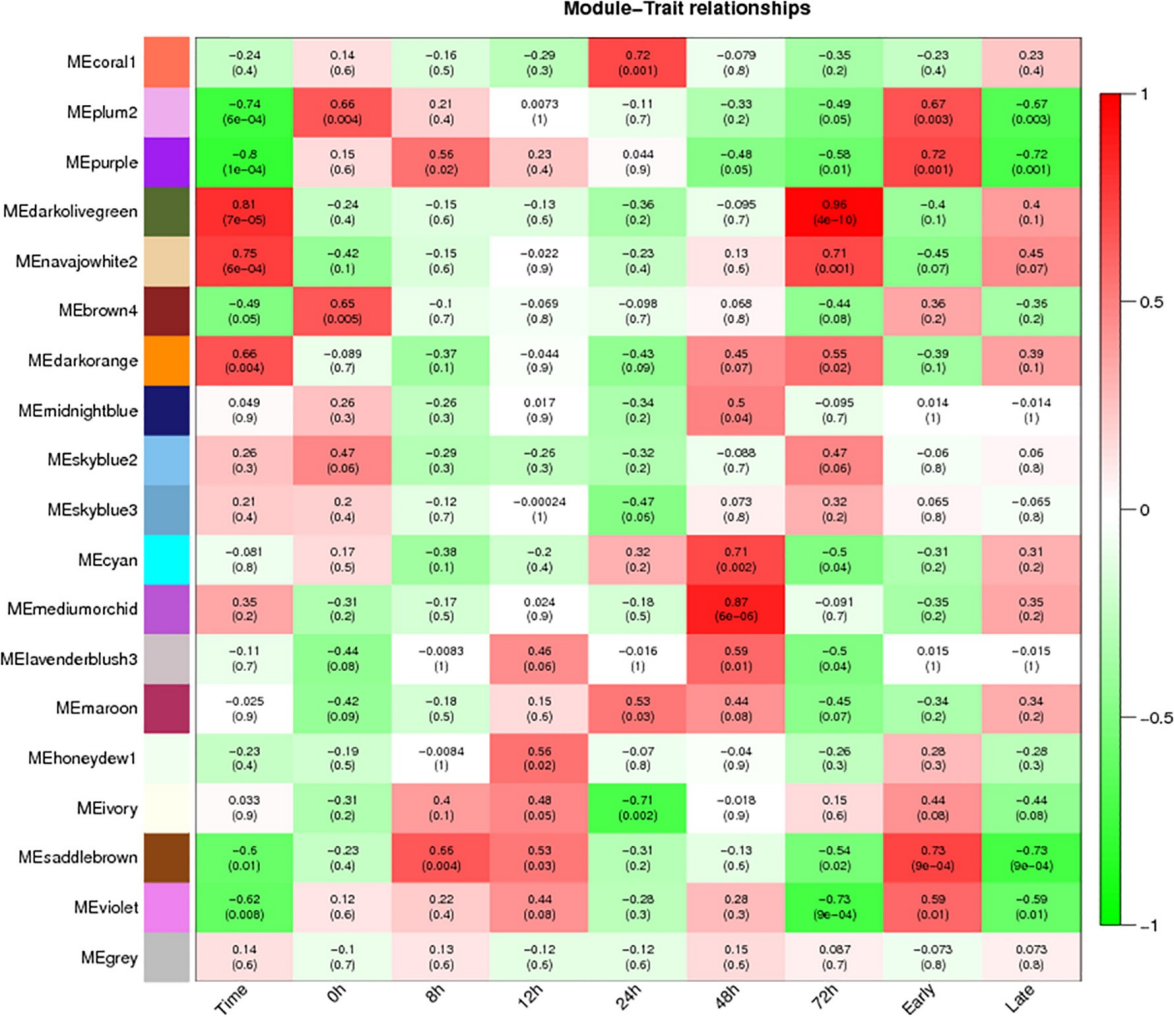
Matrix showing Module-Trait Relationships (MTRs) for Jiangcheng 3A. Each row corresponds to a module. Each column corresponds to a time result. The MTRs are colored based on their correlation: red indicates a strong positive correlation and green indicates a strong negative correlation.

**Fig 4:**
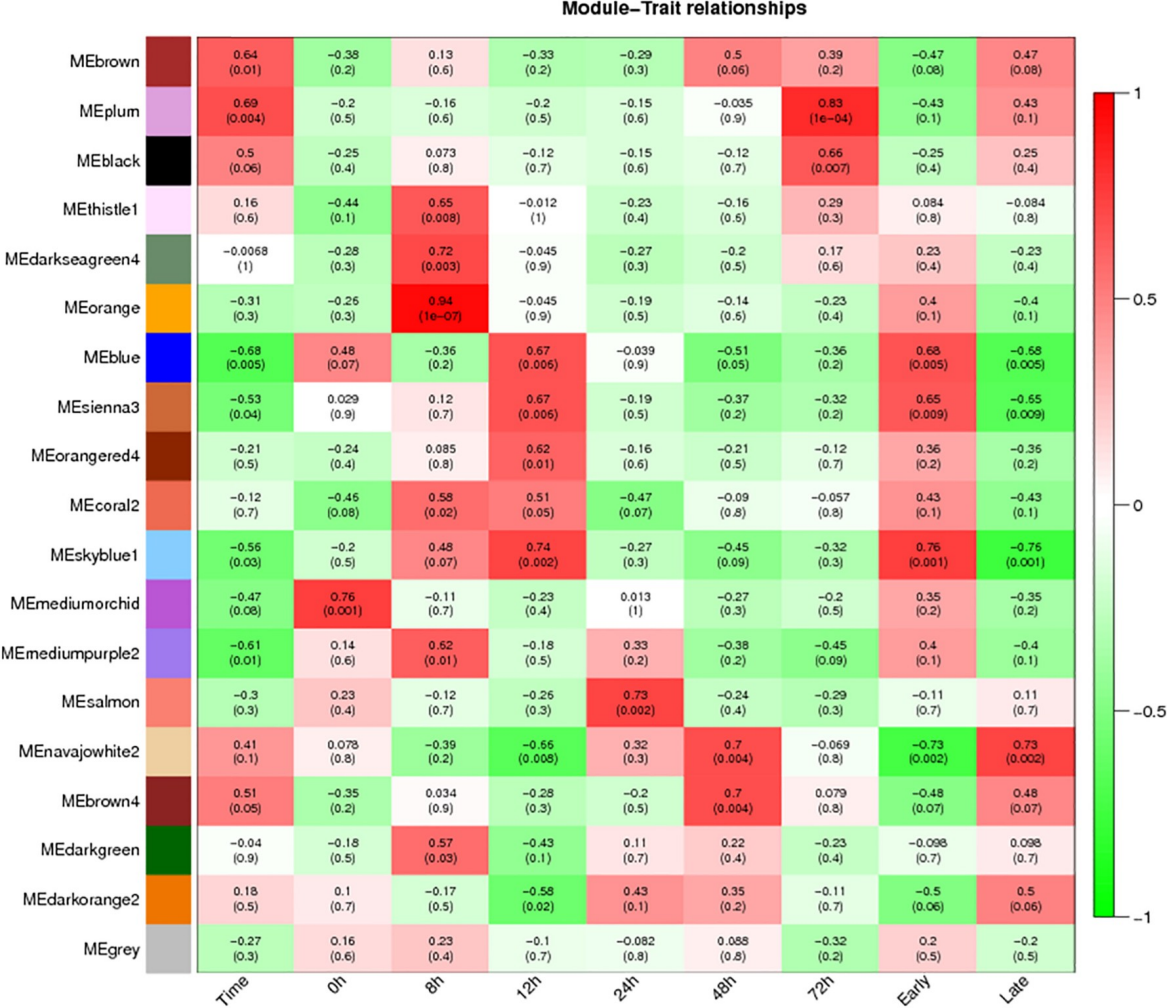
Matrix showing Module-Trait Relationships (MTRs) for 9311A. Each row corresponds to a module. Each column corresponds to a time result. The MTRs are colored based on their correlation: red indicates a strong positive correlation and green indicates a strong negative correlation.

The biomass of *T*. *horrida* in 9311A immature kernels increased by 12 h, so the 12 h immediately after *T*. *horrida* infection was key. Therefore, we also analyzed correlation with *T*. *horrida* infection at two stages, early (before 12 h) and later (after 12 h; Figs 3 and 4). The modules of association with *T*. *horrida* infection in Jiangcheng 3A and 9311A were identified. For Jiangcheng 3A, the saddlebrown (MTRs = 0.73, P = 9e-4) and purple (MTRs = 0.72, P = 0.001) modules showed high correlation with resistance at the early stage of infection. The coral (MTRs = 0.72, P = 0.001) module was highly correlated with resistance at 24 h after infection, mediumorchid (MTRs = 0.87, P = 6e-06) and cycan (MTRs = 0.71, P = 0.002) modules at 48 h, and darkolivegreen (MTRs = 0.96, P = 4e-10) and navajowhite (MTRs = 0.71, P = 0.001) at 72 h (Fig 3). For 9311A, the skyblue (MTRs = 0.76, P = 0.001) module was highly correlated with resistance at the early stage of infection, and navajowhite (MTRs = 0.73, P = 0.002) at the later stage. The darkseagreen (MTRs = 0.72, P = 0.003) and orange (MTRs = 0.94, P = 1e-07) at 8 h, skyblue1 (MTRs = 0.74, P = 0.002) at 12 h, salmon (MTRs = 0.73, P = 0.002) at 24 h, and plum (MTRs = 0.83, P = 1e-04) at 72 h showed a high correlation with *T*. *horrida* infection (Fig 4).

In order to further clarify the biological roles of each module in response to *T*. *horrida* infection, we analyzed the biological function of the disease-related modules based on GO and KEGG data. In Jiangcheng 3A, genes in the saddlebrown module were significantly enriched in diterpenoid biosynthesis, phenylpropanoid biosynthesis, glycine, serine and threonine metabolism, folate biosynthesis, porphyrin and chlorophyll metabolism, anthocyanin biosynthesis, and riboflavin metabolism according to KEGG analysis (S4 Table). Folate biosynthesis is important to the biosynthesis of lignin and alkaloids and photorespiration [37], so it is directly involved in plant resistance. Riboflavin also participates in the foundation of pathogenic disease resistance of plants [38, 39]. The mediumorchid module was significantly enriched in phenylpropanoid biosynthesis, cutin, suberine and wax biosynthesis, flavonoid biosynthesis, plant hormone signal transduction, plant-pathogen interaction, and phenylalanine metabolism, those pathways related to resistance, and fatty acid degradation, brassinosteroid biosynthesis, and tyrosine metabolism also showed significant enrichment. In a similar way, the resistance-related pathways, such as plant-pathogen interaction and plant hormone signal transduction were significantly enriched in the coral and purple modules.

In male sterile line 9311A, genes in the skyblue module exhibited significant enrichment in resistance-related pathways, such as plant hormone signal transduction, photosynthesis and carbon metabolism (S5 Table). In the darkseagreen module, most genes were involved in phenylalanine metabolism, flavonoid biosynthesis, glycine, serine and threonine metabolism, phenylpropanoid biosynthesis, ABC transporters, and tyrosine metabolism, these are resistance-related pathways. For the orange module, plant hormone signal transduction, plant-pathogen interaction, peroxisome, and cyanoamino acid metabolism were significantly enriched. The results showed that the key gene was varied in response to *T*. *horrida* infection at the different stages, and involved in different biological processes between the early and later stages. At the early stage, the resistance response was mainly related to the metabolism of physical resistance, such as lignin that could prolong the infection of the pathogen. At the later stage, the resistance response mainly related to the metabolism of system resistance, such as plant hormone signal transduction, plant-pathogen interaction, and phenylalanine metabolism.

### Hub gene selection for Jiangcheng 3A and 9311A co-expression networks

In order to found the genes that associated with resistance-*T*. *horrida*, modules with the highest correlation were selected for further analysis. For Jiangcheng 3A, we selected the saddlebrown (MTRs = 0.73, P = 9e-04) module at the early stage, and the mediumorchid (MTRs = 0.87, P = 6e-6) and darkolivegreen (MTRs = 0.96, P = 4e-10) module for the later stage, they had high correlation to disease resistance at 48 and 72 h, respectively (Fig 3). For 9311A, we chose the skyblue module (MTRs = 0.76, P = 0.001) at the early stage, the navajowhite (MTRs = 0.73, P = 0.002) module at the later stage (Fig 4).

**Fig 5:**
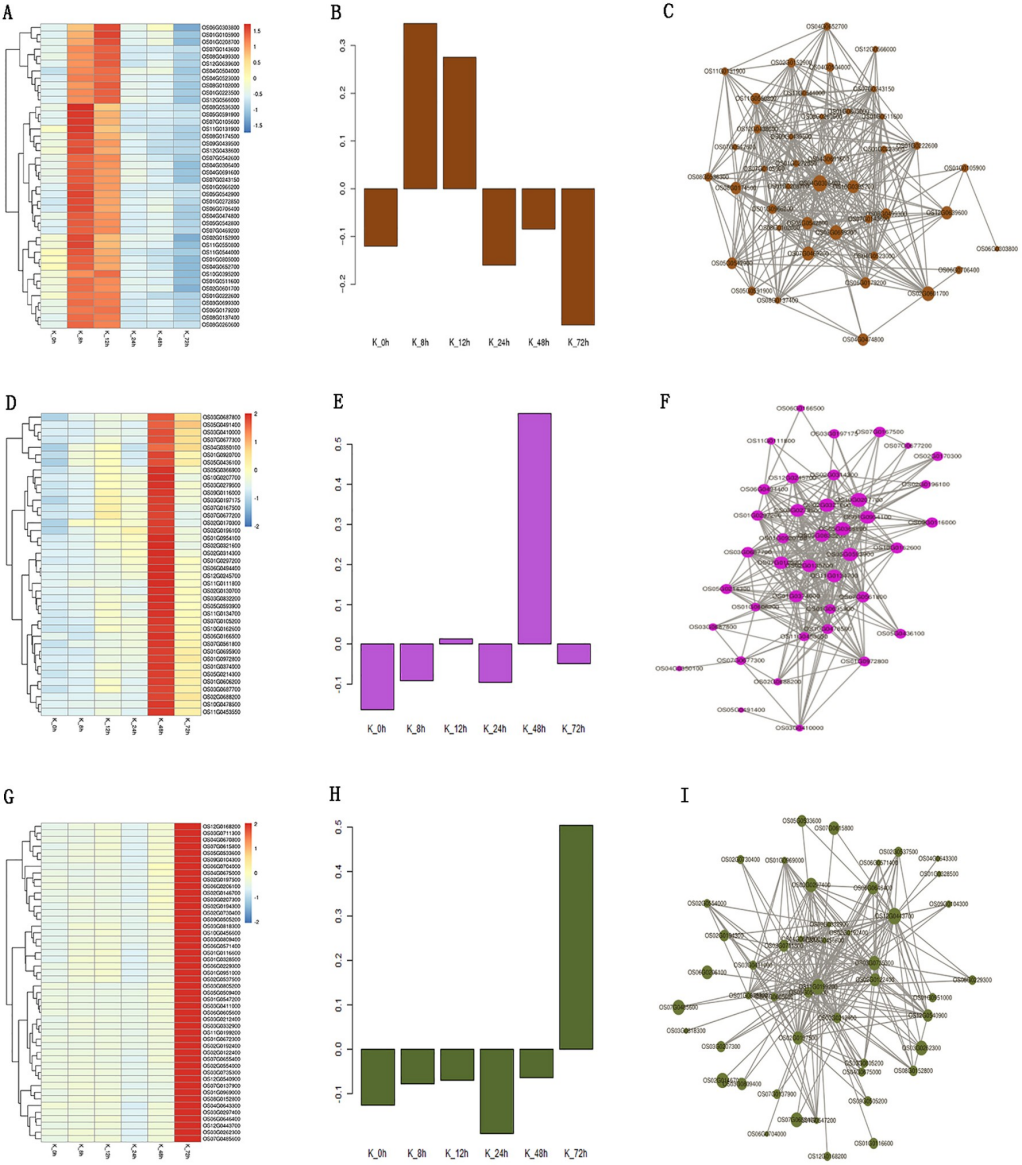
Co-expression network analysis of saddlebrown, mediumorchid, and darkolivegreen modules in Jiangcheng 3A. A, D, G Heatmaps showed the expression of genes in the saddlebrown, mediumorchid, and darkolivegreen modules, respectively. B, E, H Eigen-gene expression profiles for the saddlebrown, mediumorchid, and darkolivegreen modules at different times. The y-axis indicates the value of the module Eigen-gene and the x-axis indicates the time of sample collection. C, F, I The correlation networks corresponding to the saddlebrown, mediumorchid, and darkolivegreen modules, respectively. Candidate hub genes are shown as filled circles.

The genes in the saddlebrown module were up-regulated at the early stage in Jiangcheng 3A (Fig 5A) and the expression data of 3,045 genes in saddlebrown module shown S6 Table, eigengene expression profiles are shown in Fig 5B. The correlation network of 42 hub genes in the saddlebrown module is shown in Fig 5C and S11 Table. They include WRKY transcription factors (TFs), kinase, and CCT/B-box zinc finger protein. Other genes related to resistance, such as oxygen-evolving enhancer protein 3, DTH8, hypersensitive-induced response protein, adenylosuccinate synthetase, tetraspanin family protein, and acetyltransferase were also found. In them Os4BGlu12, polygalacturonase, DTH8, plastid-specific 50S ribosomal protein 6, and ribose-5-phosphate isomerase A were identified as candidate hub genes for this module. The genes in the mediumorchid module were up-regulated at 48 h in Jiangcheng 3A (Fig 5D) and the expression data of 726 genes in mediumorchid module shown S7 Table, eigengene expression profiles are shown in Fig 5E. Forty hub genes encoding expressed proteins were highly correlated (Fig 5F, S11 Table), including two MYB TFs Osmyb4 and Osmyb3, WRKY TFs, and bHelix-loop-helix transcription factor. The ATPase, OsVPE4, glutathione S-transferase, bHelix-loop-helix transcription factor, calreticulin precursor, OsCrll2, and OsIAA20-Auxin-responsive Aux/IAA gene family members were identified as candidate hub genes for this module. In the darkolivegreen module, the genes were up-regulated at 72 h in Jiangcheng 3A (Fig 5G) and the expression data of 8,779 genes in darkolivegreen module shown S8 Table, gene expression profiles are shown in Fig 5H. Forty-eight hub genes encoding expressed proteins were highly correlated (Fig 5I, S11 Table), including HOX1a, SDG725, CK2α3, division protein, proteasome/cyclosome repeat containing protein, PHD-finger domain containing protein, E2F family transcription factor protein, AT hook motif family protein, malonyl CoA-acyl carrier protein transacylase, inactive receptor kinase At2g26730 precursor, and CAMK_KIN1/SNF1/Nim1_like.18—CAMK including calcium/calmodulin dependent protein kinases, UBX domain-containing protein, and elongation factor 1-gamma.

**Fig 6:**
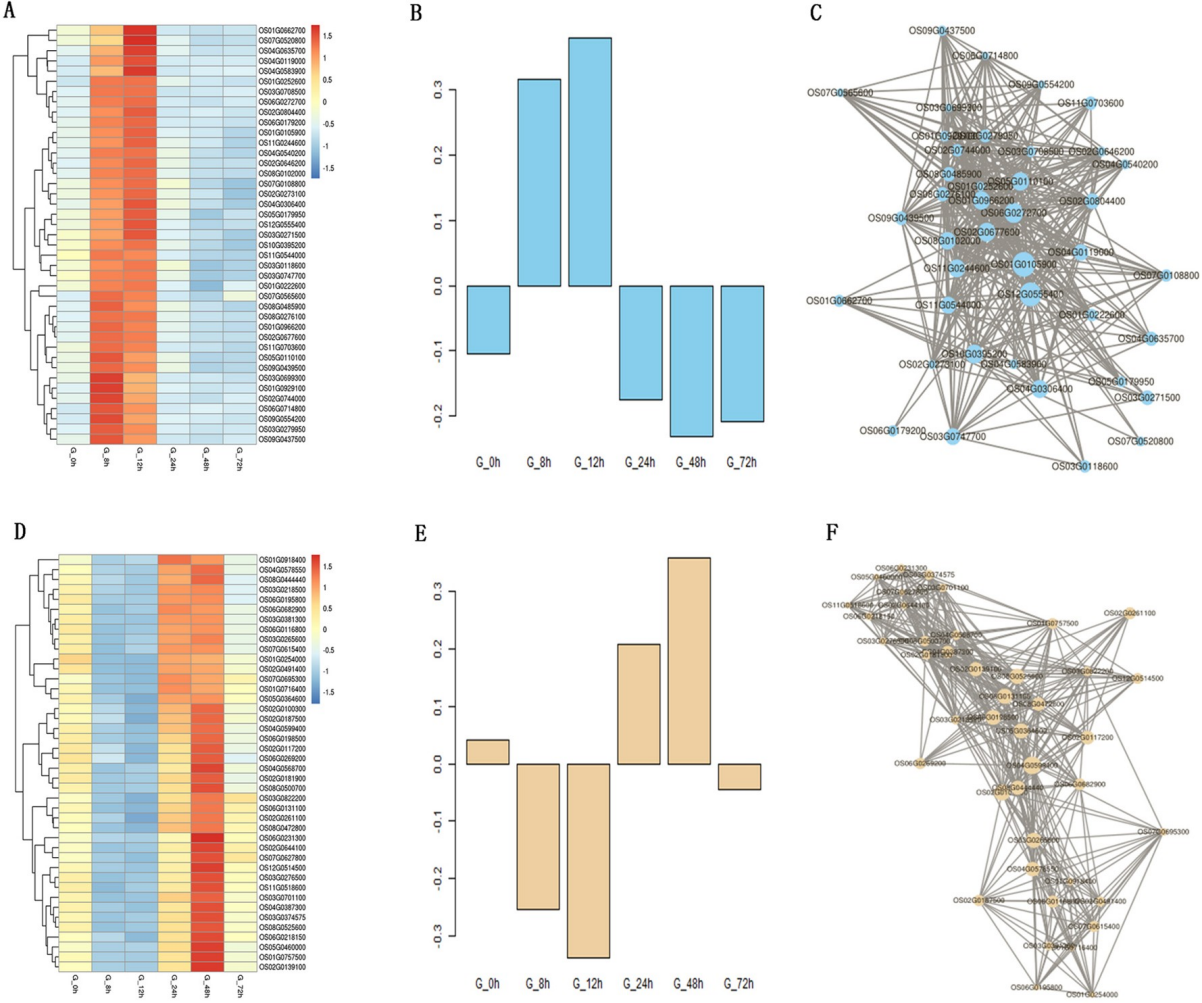
Co-expression network analysis of skyblue and navajowhite modules in 9311A. A, D Heatmaps showed the expression of genes in the skyblue and navajowhite modules, respectively. B, E Eigen-gene expression profiles for the skyblue and navajowhite modules at different times. The y-axis indicates the value of the module Eigen-gene and the x-axis indicates the time of sample collection. C, F The correlation networks corresponding to the skyblue and navajowhite modules, respectively. Candidate hub genes are shown as filled circles.

The genes in the skyblue module were up-regulated at the early stage in 9311A (Fig 6A) and the expression data of 1,480 genes in skyblue module shown S9 Table, eigengene expression profiles are shown in Fig 6B. Among them, there were 41 genes that encoded express proteins and were highly correlated (Fig 6C, S11 Table), including an MYB family transcription factor, two B-box zinc finger family proteins, YGGT family protein, uncharacterized glycosyltransferase, ANTH domain containing protein, and 17 expressed protein. OsTPKb, PsbP, UDP-glucoronosyl and UDP-glucosyl transferase domain containing protein, kinase, enoyl-CoA hydratase/isomerase family protein, and peptidyl-prolyl cis-trans isomerase were identified as candidate hub genes for this module. According to the heatmap for 9311A at the later stage (Fig 6D), genes in the navajowhite module were up-regulated and the expression data at different infection times shown S10 Table, eigengene expression profiles are shown in Fig 6E. Forty-two genes were highly correlated, including six heat shock proteins, and two integral membrane proteins (Fig 6F, S11 Table). OsHop/Sti1a, scramblase, OsHSP71.1, OsABA8ox2, and protein kinase APK1B were identified by WGCNA as candidate hub genes for this module. In addition, there were a number of expressed protein genes with unknown functions in both hub gene networks that may have important functions.

**Fig 7:**
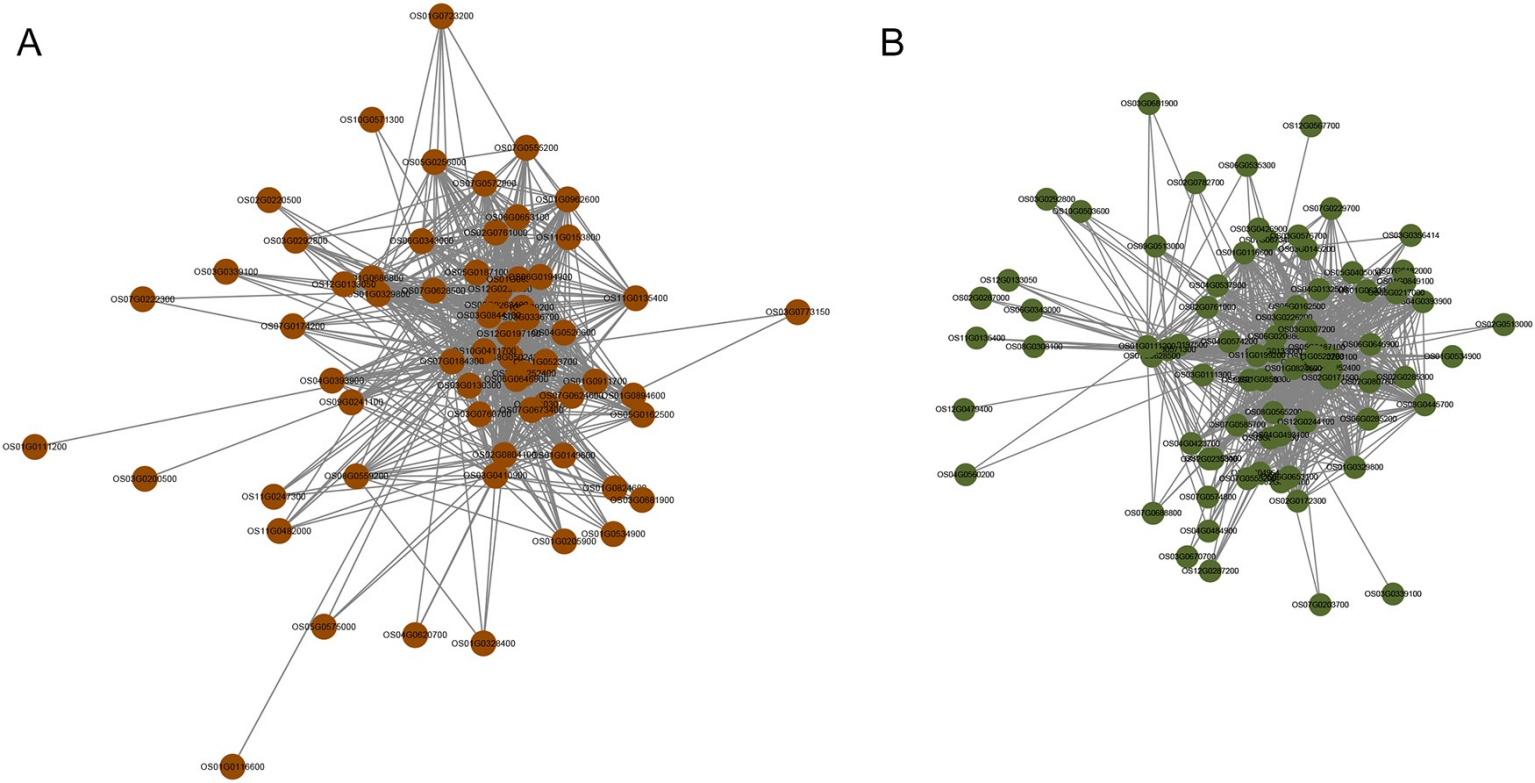
Core disease resistance gene network for Jiangcheng 3A and 9311A derived from comparing the core genes with known resistance genes.

We further analyzed the distribution of the core disease resistance gene networks that operate in RKS resistance. The results showed that common resistance genes were distributed in different modules in the two male sterile lines according to the degree of connection between co-expressed genes. For 9311A, the core disease resistance genes were mainly distributed in the brown modules (Fig 7A), which included protein kinase, LSM domain containing protein, HDA705, phosphoribosylamine—glycine ligase, OsHXK7, OsNAS2, and RSUS2. In Jiangcheng 3A, the core disease resistance genes were distributed in the darkolivegreen modules, the network of representative genes is shown in Fig 7B. Genes that had a high degree of connection included OsPBL1, protein kinase family protein, serine/threonine-protein kinase NAK, OsARF24, CTB4a, OsSRK, OsDIS1, wx, and OsDof3 TFs. Overall, in response to RKS resistance, the two male sterile lines had some common aspects, but differences were also found in the regulatory gene networks.

## Discussion

In order to identify the mechanisms involved in RKS response and search for the major modules and key genes that have important roles in the resistance of the two male sterile lines to *T*. *horrida*, we constructed the gene co-expression network. This was done using WGCNA and different modules were identified according to transcriptome data as to how the two male sterile lines were affected by *T*. *horrida* infection at different time points. Furthermore, a comprehensive view of rice plant response to *T*. *horrida* infection at the transcriptome level was obtained. The process of plant disease resistance is complex, and involves many biological signal transduction pathways and genes. Networking is an important analytical method for identifying functional genes that are involved in plant disease resistance and clarification of interactions [40–42]. In our study, pathways included in the network such as stress response, photosynthesis and carbon metabolism and other biosynthetic processes are important for plant defense against fungi. Therefore, network analysis helped to identified hub genes and major modules related to disease resistance.

We selected several modules for further analysis, and gene biological functions that were included in those modules of the two genotypes were explained based on GO and KEGG analyses. In both Jiangcheng 3A and 9311A, plant-pathogen interaction, phenylalanine metabolism, and plant hormone signal transduction were common metabolic pathways involved in resistance, the pathways were more enriched in Jiangcheng 3A than in 9311A. In addition, plant-pathogen interaction, phenylalanine metabolism, and plant hormone signal transduction were shown to be crucial in the overall resistance process. For example, previous studies have shown that phenylalanine metabolism produced many secondary metabolites associated with plant disease resistance, such as lignins, salicylates, coumarins, flavonoids, and phytoalexins [43–45]. Intracellular calcium transients participated in some biological processes associated with resistance, such as oxidative burst, PR gene expression, hypersensitive cell death, and systemic acquired resistance during plant-pathogen interactions [46–48]. The pathway of phenylalanine metabolism produced the enzymes phenylalanine ammonia-lyase (PAL) and was key to salicylic acid (SA) biosynthesis, SA is more important than jasmonic acid in defence response against biotrophic fungi [49].

Folate biosynthesis showed a significant accumulation in Jiangcheng 3A at the early stage, but this was not found in 9311A. The precursor of para-aminomethyl- benzoic acid (Paba) that forms as an intermediate metabolite in the process of folate biosynthesis, is a branched acid, which can participate in the synthesis of lignin though the formation of phenylalanine. The pathway of phenylalanine metabolism showed a significant accumulation in Jiangcheng 3A at 48 h. This result demonstrated that primary metabolism is involved in the resistance process at the beginning of *T*. *horrida* infection and that SA plays an important role at 48 h and thereafter. Other pathways related to resistance, such as flavonoid biosynthesis and cutin, suberine and wax biosynthesis also accumulated in Jiangcheng 3A at 48 h. With further spread of the pathogen in the rice kernel, the related disease resistance processes appeared to form a complex, three-dimensional resistance network. Overall, gene expression analyses revealed notable alterations in the transcriptional levels of genes related to plant metabolism, suggesting some roles of primary host metabolism in relation to defense mechanisms.

The gene networks we constructed showed that there are large differences between Jiangcheng 3A and 9311A at different stages of infection. The hub genes and their degree of connection in the two varieties varied, in Jiangcheng 3A, DTH8 had a high degree of connectivity in the network. *T*. *horrida* is a biotrophic lifestyle fungi and infects rice floral organs, so Jiangcheng 3A could hinder the infection by delaying flowering. Hypersensitivity has an important function in plant resistance to biotrophic fungi [50]. The hypersensitive-induced response protein was found in the hub gene network. In 9311A, some of the genes associated with stress resistance showed higher connectivity at different infection stages, such as PsbP at the early stage, it has a key role in photosynthesis and is a crucial in the early stages of the overall resistance process, and OsHop/Sti1a at the later stage, previous research has shown that OsHop/Sti1a plays an important role in rice blast resistance [51].

Transcription factors (TFs) including MYB, WRKY, and bHelix-loop-helix TF family members [52–54], play a key role in plant defense responses [55]. In our study, there were several TFs in hub genes, such as OsWRKY17 TFs. WRKY TFs have important roles in plant responses to pathogen infection [56], and more than half of the 103 WRKY TF genes present in the rice genome are differentially expressed under biotic and abiotic stress conditions [56,57]. In addition, several MYB TFs are also found in hub genes, which mainly regulate secondary metabolism, such as hormone signal transduction, phenylpropanoid metabolism, and responses to biotic and abiotic stressor [58, 59]. So, our clarification of TF mechanisms involved in resistance processes will not only lead to the understanding of signal transduction networks through controlling TFs, but will also lead to the discovery of new resistance-related genes.

In plant defense response processes, the key known resistance genes are important markers. In our study, several protein kinases, such as OsPBL1, OsSRK, CTB4a and serine/threonine-protein kinase NAK, had high connectivity in networks of key resistance genes in Jiangcheng 3A and 9311A. Protein kinase have an important role in the regulation of signal perception, transmission, the gene expression and many other physiological processes in regulating plant growth and defense response [60]. MAPKKK serine/threonine-protein kinase is involved in the regulation of a MAP kinase cascade that negatively regulates SA dependent defense responses, abscisic acid (ABA) signaling, and ethylene-induced senescence [61]. Lee et al. identified that the serine/threonine protein kinase OsPBL1 is a key resistance gene to rice stripe disease, and that it is regulated downstream from the actions of cytokinin and SA [62]. Other genes related to resistance, such as wx, OsDof3, and OsARF24 were also found in the core gene network (Fig 7).

## Supporting information

S1 Table Specific primers of differential gene sequences for qRT-PCR. (XLSX)

S2 Table Core resistant gene in Jiangcheng 3A. (XLSX)

S3 Table Core resistant gene in 9311A. (XLSX)

S4 Table Overview of the significantly overrepresented KEGG pathways associated with the modules detected using WGCNA in Jiangcheng 3A. (XLSX)

S5 Table Overview of the significantly overrepresented KEGG pathways associated with the modules detected using WGCNA in 9311A. (XLSX)

S6 Table All gene list and expression of saddlebrown module in Jiangcheng 3A. (XLSX)

S7 Table All gene list and expression of mediumorchid module in Jiangcheng 3A. (XLSX)

S8 Table All gene list and expression of darkolivegreen module in Jiangcheng 3A. (XLSX)

S9 Table All gene list and expression of skyblue module in 9311A. (XLSX)

S10 Table All gene list and expression of navajowhite module in 9311A. (XLSX)

S11 Table Hub gene list of Saddlebrown, Mediumorchid, Darkolivegreen, Skyblue, and Navajowhite module in Jiangcheng 3A and 9311A. (XLSX)

S1 Fig Power values was selected in 9311A. (TIF)

S2 Fig Power values was selected in Jiangcheng 3A. (TIF)

S3 Fig qRT-PCR validation of selected genes in Jiangcheng 3A and 9311A after *T*. *horrida* inoculation. (TIF)
