# Supplementary figures and images for: Comparison of gene co-networks analysis provide a systems view of rice (Oryza sativa L.) response to Tilletia horrida infection
Source: PLoS One. 2018 Oct 29;13(10):e0202309. doi: 10.1371/journal.pone.0202309 (PMC6205584; doi:10.1371/journal.pone.0202309)

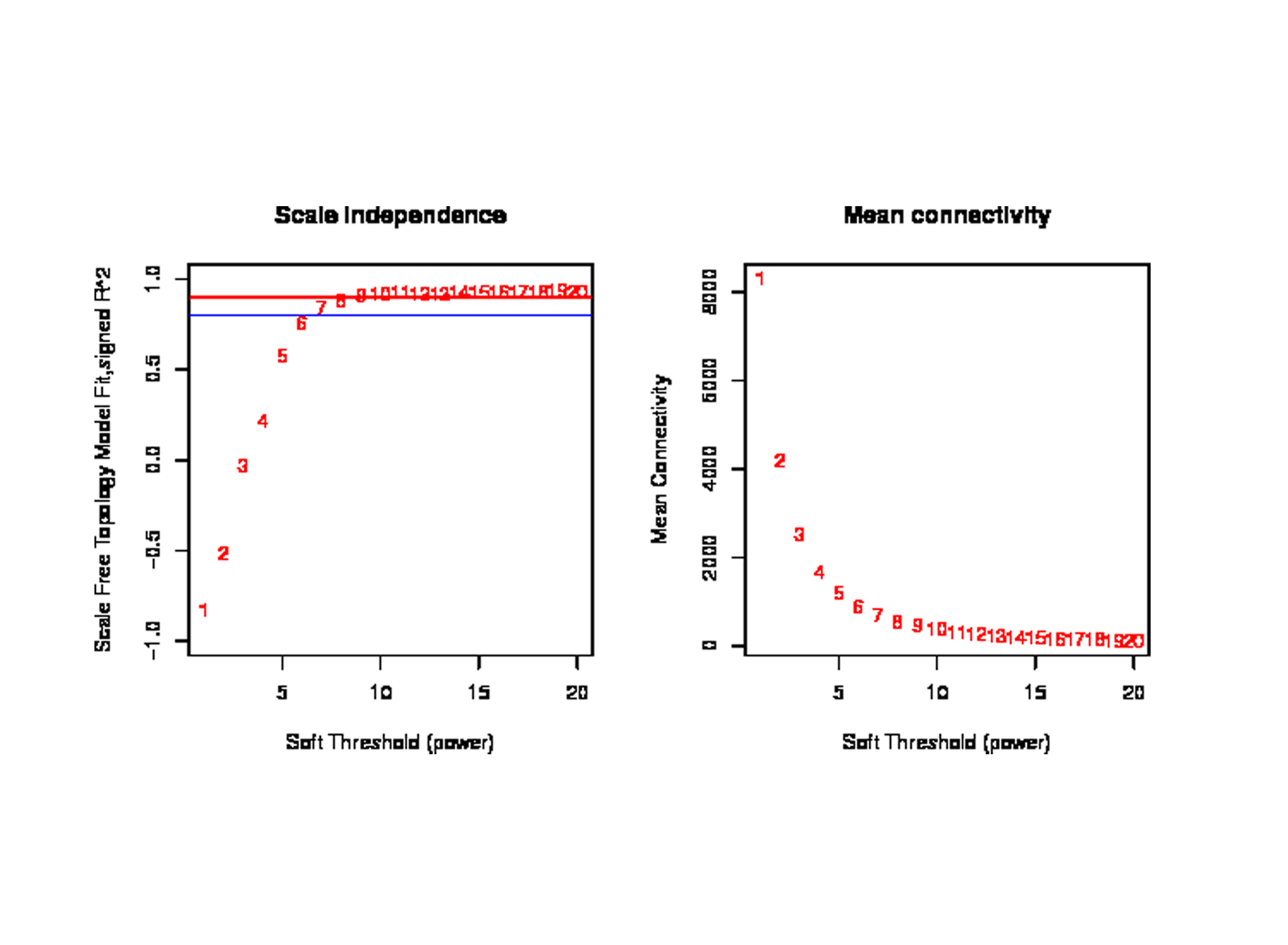

Supplement: S1 Fig — (TIF) [file pone.0202309.s012.tif]

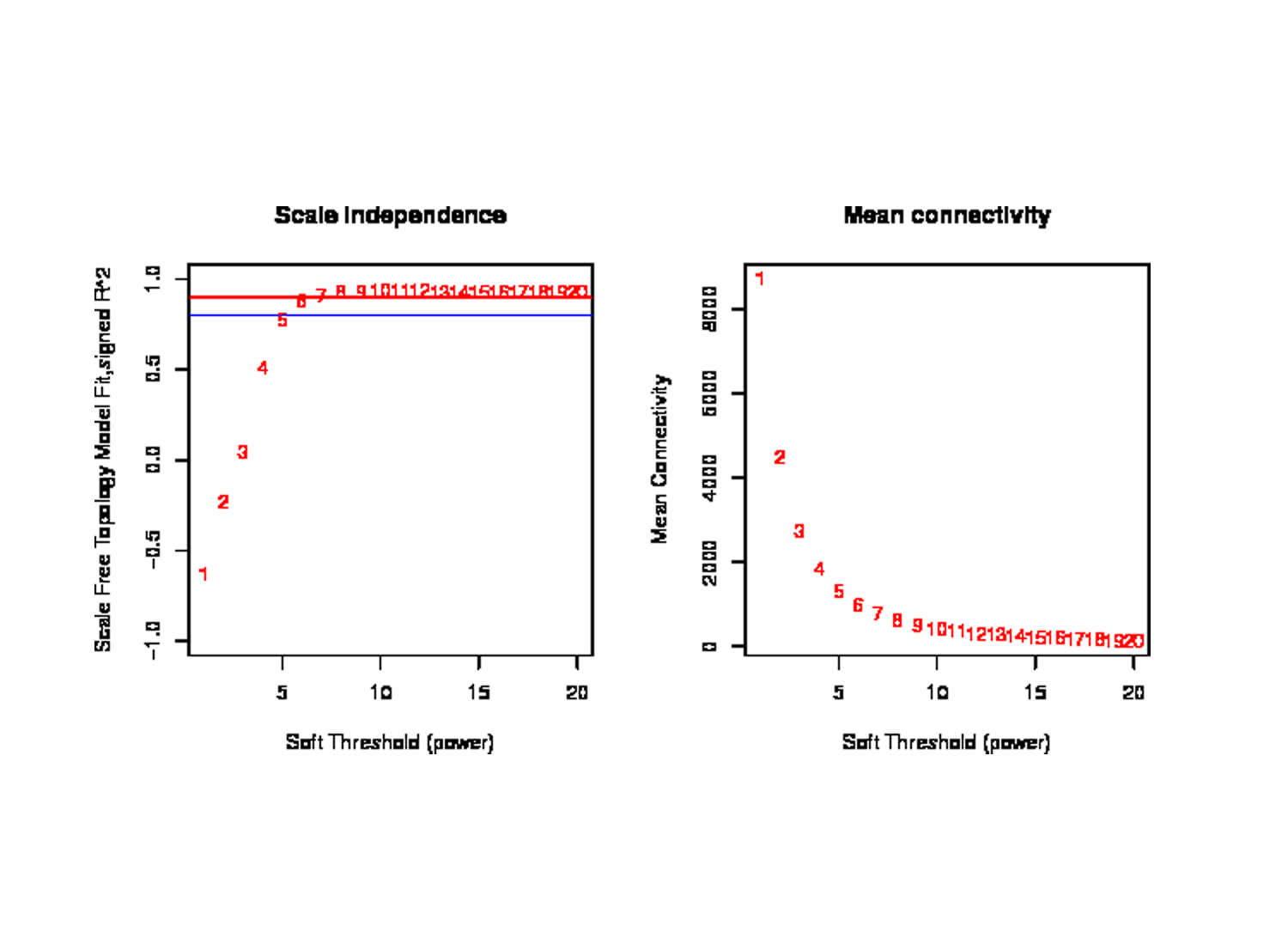

Supplement: S2 Fig — (TIF) [file pone.0202309.s013.tif]

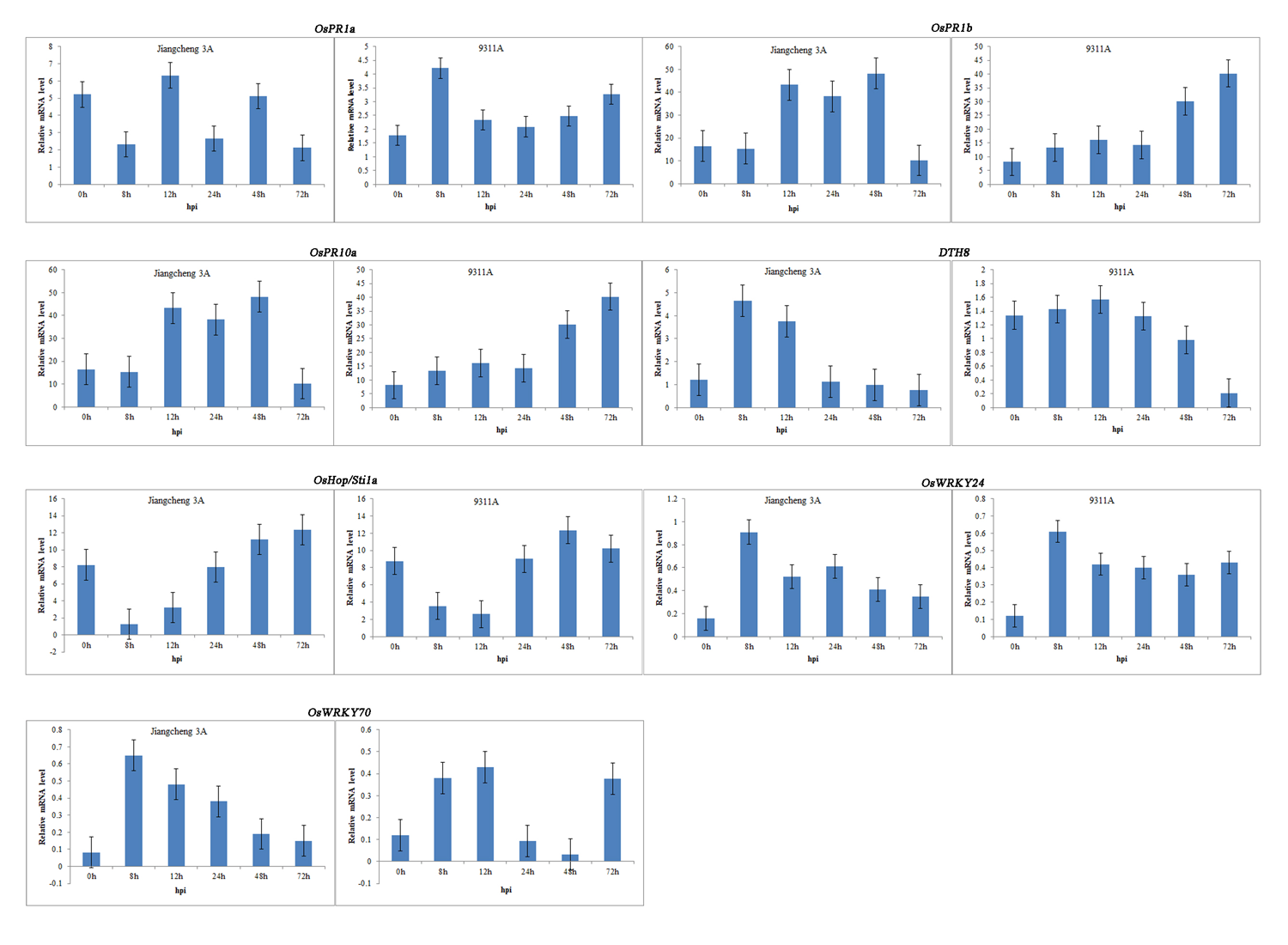

Supplement: S3 Fig — (TIF) [file pone.0202309.s014.tif]
